# Performance of natural-dye-sensitized solar cells by ZnO nanorod and nanowall enhanced photoelectrodes

**DOI:** 10.3762/bjnano.8.31

**Published:** 2017-01-30

**Authors:** Saif Saadaoui, Mohamed Aziz Ben Youssef, Moufida Ben Karoui, Rached Gharbi, Emanuele Smecca, Vincenzina Strano, Salvo Mirabella, Alessandra Alberti, Rosaria A Puglisi

**Affiliations:** 1 Laboratoire des Semi-conducteurs et Dispositifs Electroniques, LISIER, University of Tunis, Ecole Nationale Supérieure d’Ingénieurs de Tunis, 05 Av. Taha Hussein 1008 Montfleury, Tunis, Tunisia; 2 Laboratoire de Photovoltaïque, centre de Recherche et des Technologies de l’énergie, Technopole de Borej-Cedria, BP 95, Hammam-Lif, Tunis 2050, Tunisia; 3 CNR-IMM, Zona Industriale Strada VIII, N°. 5, 95121 Catania, Italy; 4 MATIS CNR-IMM and Dipartimento di Fisica e Astronomia, Università di Catania, via S. Sofia 64, Catania, Italy

**Keywords:** DSSCs, *I*–*V* measurement, nanorods, nanowalls, natural dye, ZnO

## Abstract

In this work, two natural dyes extracted from henna and mallow plants with a maximum absorbance at 665 nm were studied and used as sensitizers in the fabrication of dye-sensitized solar cells (DSSCs). Fourier transform infrared (FTIR) spectra of the extract revealed the presence of anchoring groups and coloring constituents. Two different structures were prepared by chemical bath deposition (CBD) using zinc oxide (ZnO) layers to obtain ZnO nanowall (NW) or nanorod (NR) layers employed as a thin film at the photoanode side of the DSSC. The ZnO layers were annealed at different temperatures under various gas sources. Indeed, the forming gas (FG) (N_2_/H_2_ 95:5) was found to enhance the conductivity by a factor of 10^3^ compared to nitrogen (N_2_) or oxygen (O_2_) annealing gas. The NR width varied between 40 and 100 nm and the length from 500 to 1000 nm, depending on the growth time. The obtained NWs had a length of 850 nm. The properties of the developed ZnO NW and NR layers with different thicknesses and their effect on the photovoltaic parameters were studied. An internal coverage of the ZnO NWs was also applied by the deposition of a thin TiO_2_ layer by reactive sputtering to improve the cell performance. The application of this layer increased the overall short circuit current *J*_sc_ by seven times from 2.45 × 10^−3^ mA/cm^2^ to 1.70 × 10^−2^ mA /cm^2^.

## Introduction

**Figure 1:**
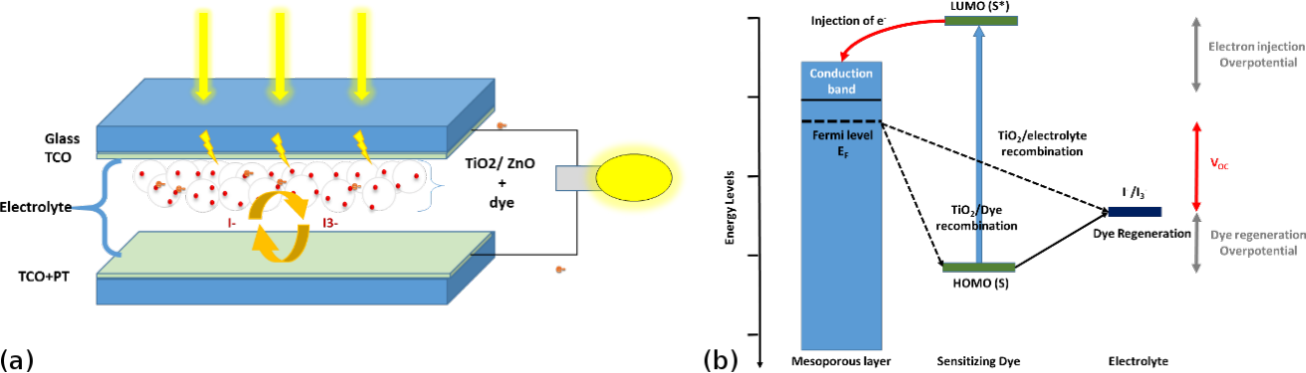
(a) Principle of operation and (b) energy level diagram of a typical DSSC.

Energy demand has increased rapidly during the last forty years to reach a growth rate of 1.8% per year [1]. To satisfy this growing need, it is necessary to find new sources of renewable energy. For instance, photovoltaic (PV) technologies offer a promising green industry for the future power demand. Among these technological resources, dye-sensitized solar cells (DSSCs) have shown good performance since their first demonstration by O'Regan and Grätzel in 1991 [2]. Figure 1a shows the standard structure of DSSC: The first part of this structure represents the photoelectrode, composed of a wide band gap semiconductor thin layer coated on transparent conducting oxide (TCO) films. A mesoporous film layer is used as a semiconductor in the photoelectrode. Because of its stability and easy synthesis, titanium dioxide (TiO_2_) is mostly used as the semiconductor in DSSCs [2–5]. Besides, the TiO_2_ offers high electronic mobility for photogenerated electron collection, a suitable band gap, which adapts to the injection of the electrons of most studied dyes, and high surface area to enhance the dye loading by anchoring the dye [6–7].

Zinc oxide (ZnO) has been studied as a mesoporous wide band gap semiconductor for use in DSSCs. It presents itself in the form of different morphological nanostructures, such as nanorods, nanocrystals, nanowires, nanotubes and nanowalls that can be exploited to optimize the dye loading [6–9]. The main purpose of the photoelectrode is to collect the injected electrons from the excited dye absorbed by the semiconductor layer and to transport the electrons to the external load. The second main part of the structure is the counter electrode formed by a thin layer of platinum coated on TCO to accelerate the redox reaction with the electrolyte solution, which constitutes the third main part of the cell [3].

The internal process starts with the excitation of the sensitizer (S) through the absorption of a photon to obtain an excited sensitizer (S^*^). The latter injects an electron into the conduction band of the semiconductor. Then, it stays in the oxidized state (S^+^), as mentioned in Figure 1b. The injected electron reaches the external load through the semiconductor and the TCO layers. When arriving at the counter electrode, the electron reduces the redox solution and regenerates the sensitizer to complete the reaction [2,10]. Different high-raked commercial dyes, such as N719 or N3, have been widely studied in the literature and their performance has been proven. Because these organometallic dyes contain environmental pollutants, such as heavy metals [6], recent studies have focused on finding a metal-free organic dye with high performance [8,11–13]. Indeed, several studies were carried out using natural dyes, such as spinach and red turnip as a sensitizer. However, their efficiency is lower than that of commercial organometallic dyes. In this work, we investigate two different natural dyes extracted from henna and mallow plants. We discuss also their application to different semiconductor structures.

The photoelectrode is regarded as an important part in the DSSC where it represents the electron generator of the cell. Solar cell parameters, such as open-circuit voltage (*V*_oc_), short-circuit current density (*J*_sc_) and fill factor (FF), are the most significant parameters used to evaluate the enhancement of the power conversion efficiency (PCE) of solar cells [10]. The short circuit current density, *J*_sc_, depends essentially on dye adsorption, the collection efficiency in the semiconductor thin layer film and the efficiency of the collected dye molecules [3,14–15]. The adsorption of the dye can be improved by various means, such as increasing the thickness and/or the porosity of the photoelectrode or by using organized structures, such as nanowalls or nanorods. The *V*_oc_ can be improved by modifying the energy difference between the Fermi level (*E*_F_) of the semiconductor potential and redox potential (*E*_redox_) of the electrolyte [10].

## Results and Discussion

### Dye analysis

**Figure 2:**
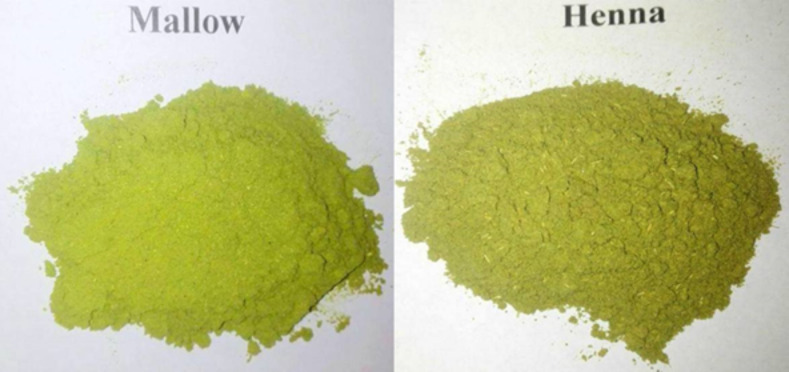
Photograph of mallow and henna powders.

In order to understand the structure of natural dye molecules and to determine the main elements responsible for absorbing light, we used FTIR spectroscopy techniques and ultraviolet–visible spectrophotometry (UV–vis) to characterize dyes extracted from henna and mallow powder as shown in Figure 2.

**Figure 3:**
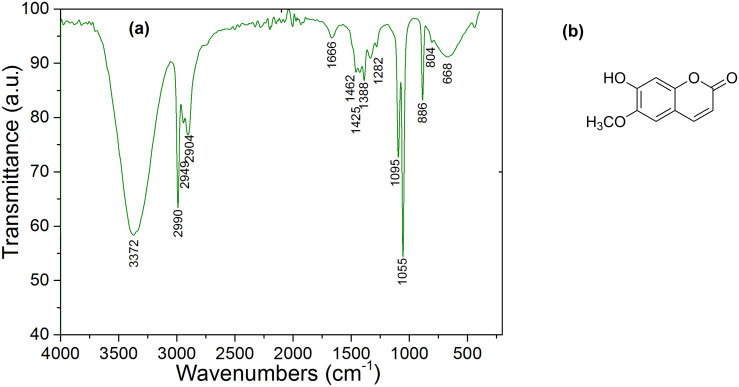
(a) FTIR spectrum of mallow. (b) Scopoletin molecule.

The scopoletin molecule, shown in Figure 3b [15–17], is the major component in the mallow dye. From the FTIR spectrum of the mallow dye (Figure 3a), it is clear that mallow has a primary O–H bond associated with the peak at 3372 cm^−1^. We notice also the existence of aliphatic groups, CH_2_ and CH_3_ (2904 cm^−1^, 2949 cm^−1^, 2990 cm^−1^), an ether function (*V*_C–O_) at 1055 cm^−1^, and a regular deformation band δC_sp2-H_ at 668 cm^−1^.

**Figure 4:**
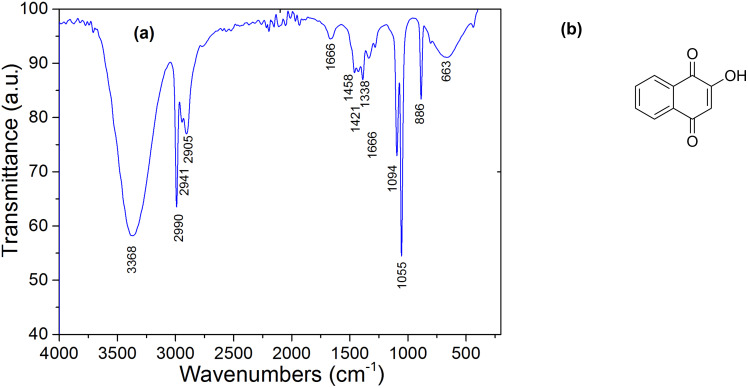
(a) FTIR spectrum of henna. (b) Lawsone molecule.

2-Hydroxy-1,4-napthaquinone (C_10_H_6_O_3_), frequently called lawsone [15,18], is one of the constituents of the natural dye henna in addition to other compounds, such as gallic acid, sterols, resin, tannin and coumarins [19]. Lawsone (Figure 4b), the main component of henna extract, has been used as a dye in the cosmetics industry. The most significant feature of the lawsone molecule is its ability to absorb visible light between 400 and 600 nm.

The FTIR spectrum of henna extract, presented in Figure 4a, shows the existence of three valence bands associated with C=C aromatics at 1338 cm^−1^, 1421 cm^−1^ and 1458 cm^−1^, and also a stretching absorption band at 3368 cm^−1^ corresponding to the vibration of the associated O–H bond.

**Figure 5:**
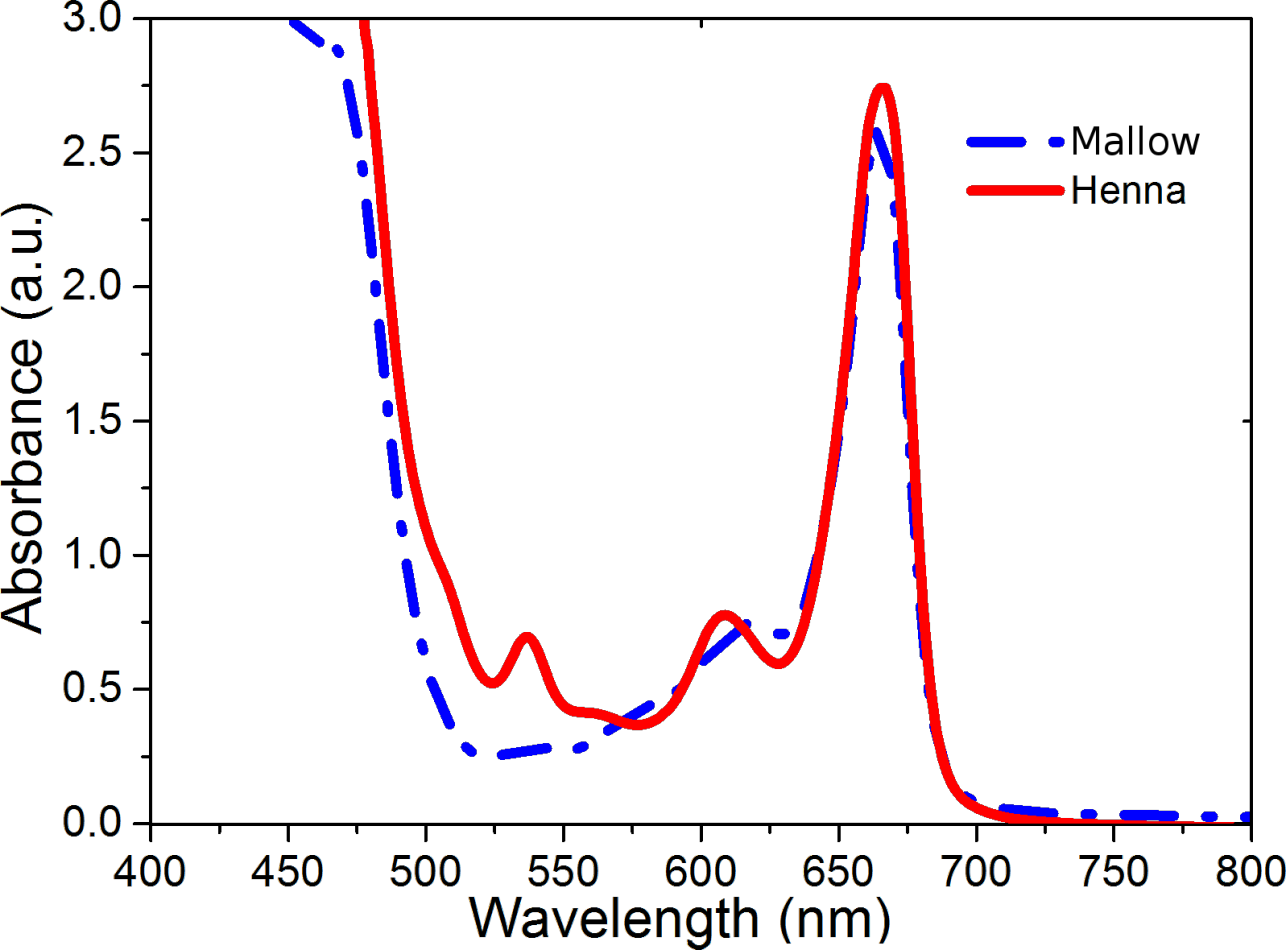
UV–vis measurements of henna and mallow dye solutions.

The UV–vis measurements of the extracted dyes of henna and mallow powders diluted in ethanol are given in Figure 5. Both of the studied dyes show two remarkable peaks: the first around 470 nm (blue light), and the second at 665 nm (red light). Both peaks correspond to the mixture of chlorophyll [20]. In the region from 525 nm to 625 nm, the mallow dye spectrum reveals two small peaks at 536 nm and 608 nm. These peaks could serve to increase the charge-transfer reaction under sun illumination in the final DSSC [21].

### Morphological results of the layers

In order to estimate the capacity of the dye loading, scanning electron microscopy (SEM) analysis was carried out to study the porosity and the morphology of the layers prepared by the CBD technique [8,22].

**Figure 6:**
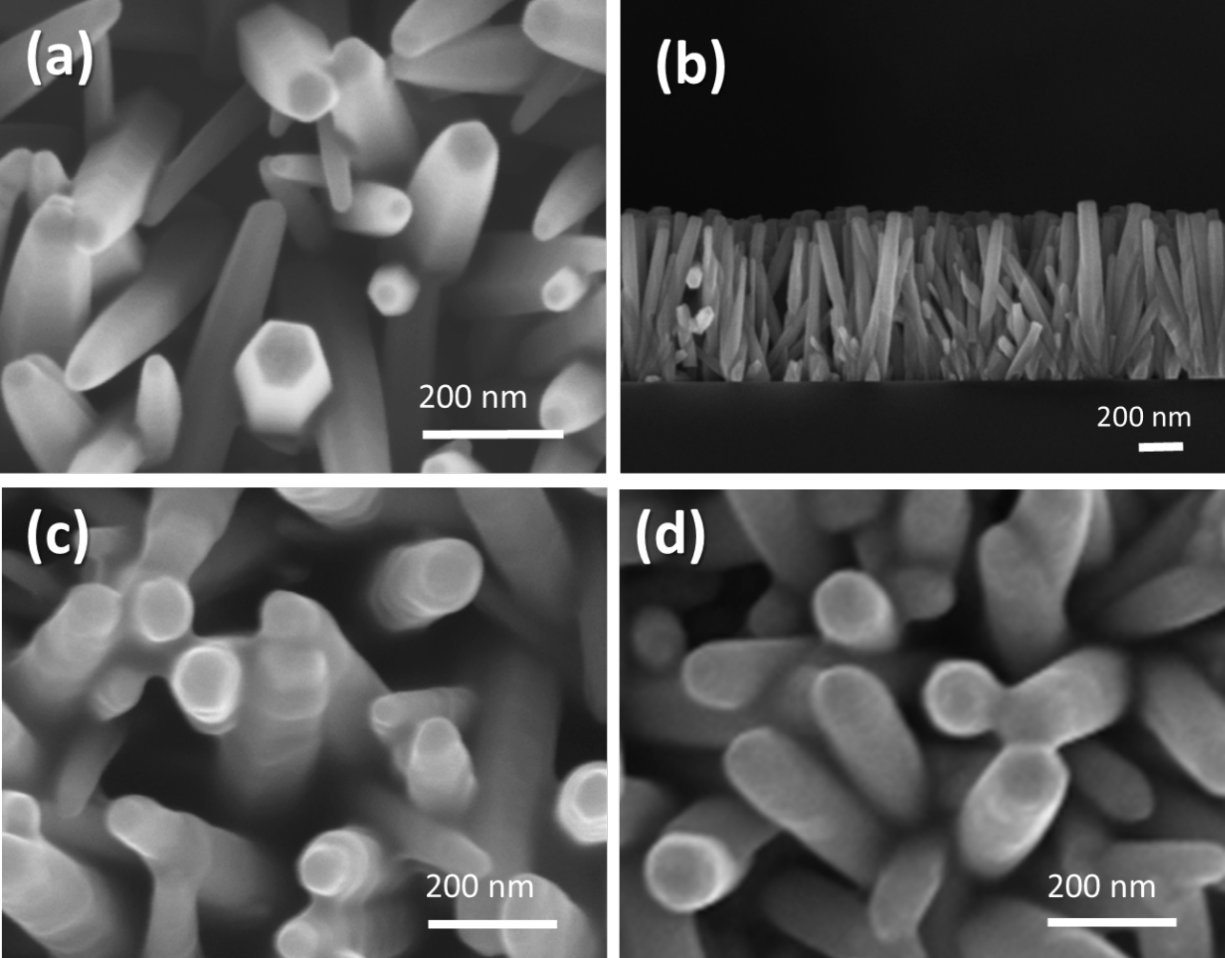
SEM images of ZnO NRs (a) before immersion in dye, (b) cross-section view of ZnO NRs, (c) after immersion in henna dye and (d) after immersion in mallow dye.

Figure 6a gives the SEM images illustrating the morphology of the ZnO NRs annealed in forming gas (FG) (N_2_/H_2_, 95:5). From this figure, we observe that nanorods cover the entire surface of the fluorine-doped tin oxide (FTO) conductive glass and have a hexagonal shape with uniform size and length [23]. The width of the NRs varies between 40 and 100 nm, while their length ranges from 500 to 1000 nm, depending on growth time, as shown in Figure 6b and mentioned by Iwu et al. [8]. In Figure 6c and Figure 6d, we give the SEM images of the ZnO NR after being immersed in henna and mallow dye, respectively. The images show the formation of a thin layer between the NRs. This layering effect may be due to a reaction between the ZnO nanoparticles and the solvent in the dye solution. From these images, we notice that the number of individual hexagonal NRs is reduced and their shapes are no longer visible. This morphological change can be explained by aggregation of the dye and Zn^2+^ when the films were immersed in henna and mallow dye [24].

**Figure 7:**
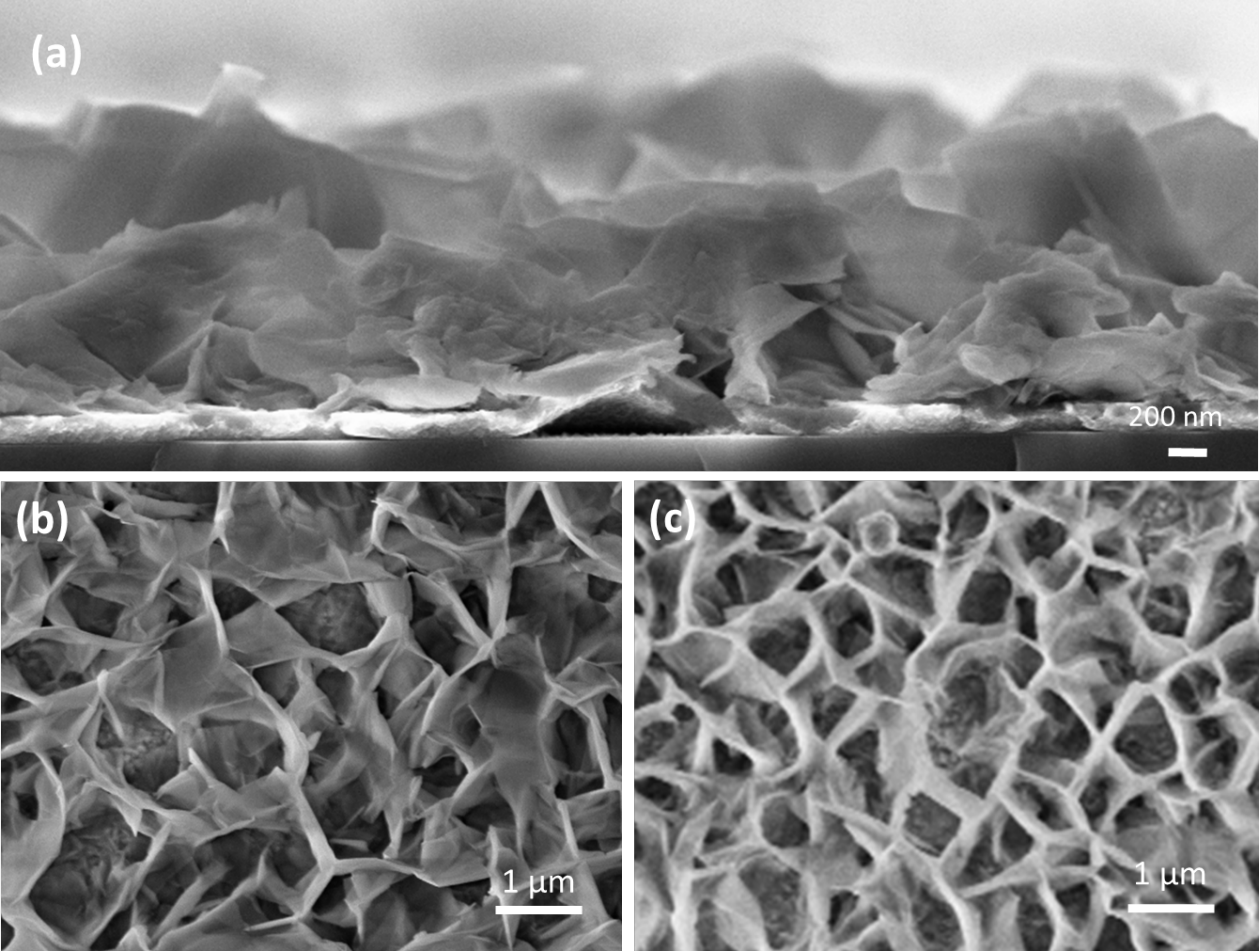
SEM images of ZnO NWs (a) cross-section view of ZnO NWs (b) before immersion in dye and (c) after immersion in mallow dye.

Figure 7a shows the morphology of the ZnO NWs annealed in FG. The NWs intersect at different angles, creating hollow spaces, acting to increase the porosity of the layer, which may improve the dye loading as observed by Polkoo et al. [25]. By analyzing the cross-section of the ZnO NWs, we obtained a NW vertical length of around 850 nm, as shown in Figure 7b. In Figure 7c, we give the images of the ZnO NWs after being immersed in mallow dye. We notice that dye molecules cover the NWs without affecting their overall shape, as compared to NRs. The formed layer does not appear clearly in the surface of the NWs. However, there is a small difference in the intersection angles between walls. After the immersion, Figure 7c shows less acute angles of wall intersections compared to those presented in Figure 7b. This variation in the angle sharpness shows that a thin layer is formed in the different intersections and not in the walls.

**Figure 8:**
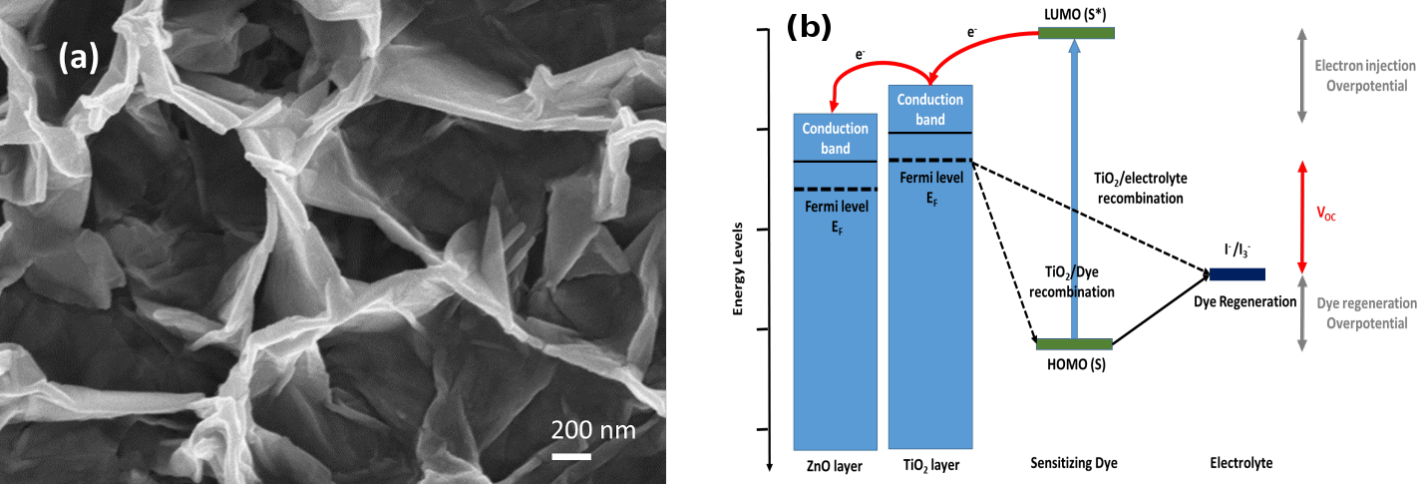
(a) SEM images of ZnO NWs after the deposition of the TiO_2_ layer by sputtering. (b) Energy level diagram of a ZnO/TiO_2_ DSSC.

Figure 8a illustrates the magnified image of the ZnO NWs over which a TiO_2_ layer was deposited by the sputtering technique. We observe that the nanowall vertical length is about 1000 nm. Besides, the shape is arranged in a more vertical orientation as compared to the nanowalls without the TiO_2_ blocking layer. The main idea is to cover the ZnO NWs with a thin layer of TiO_2_ (about 150 nm of thickness), which enhances the electron transport by exploiting the high mobility and diffusion rate of the ZnO. This covering layer also increases the efficient electron diffusion from the TiO_2_ layer to the ZnO NWs. Using this structure, the electrons were quickly transported from the sensitizer to the ZnO through the TiO_2_ to reach the FTO (Figure 8b). This solution created an energy barrier allowing the reduction of the charge recombination [26].

### Optical characterization

Based on the SEM analysis, we conclude that the growth time can influence the layer thickness, which affects the light absorbance [9] and conductivity [27]. For these reasons, after the growth of the ZnO NR layers on FTO, they were annealed in O_2_, FG and N_2_ gas at 300 °C.

As shown in Table 1, the sheet resistance of the ZnO NRs depends on the used annealing gas. By using O_2_, we obtained the highest sheet resistance with a ratio of 10^3^ compared to those prepared under N_2_ or FG. The best sheet resistance was measured in the case of annealing in FG.

**Table 1:** Sheet resistance of ZnO layers for different ambient annealing gases and *J*–*V* measurement results of the assembled cells.

Cell N°	Dye	Photoanode layer	Annealing gas	Annealing temp. (°C)	Sheet resistance (Ω/sq)	Growth time (min)	*J*_SC_ (mA/cm^2^)	*V*_OC_ (V)	FF (%)

1	henna	ZnO nanorods	FG	200	5.3 × 10^3^	90	1.26 × 10^−1^	0.370	27
2	mallow		FG			90	1.55 × 10^−1^	0.425	27
3			FG			45	1.49 × 10^−1^	0.428	29
4			N_2_		90 × 10^3^	90	5.35 × 10^−2^	0.136	30
5			as deposited	–	600 × 10^3^	90	4.24 × 10^−2^	0.379	27
6			O_2_	200	1.4 × 10^6^	–	–	–	–
7		TiO_2_ + ZnO nanowalls	FG	300	5.3 × 10^3^	5	8.02 × 10^−3^	0.426	34
8			FG	200			1.70 × 10^−2^	0.380	26
9			as deposited	–	600 × 10^3^		1.23 × 10^−2^	0.413	29
10		ZnO nanowalls	FG	200	5.3 × 10^3^		2.45 × 10^−3^	0.169	29

**Figure 9:**
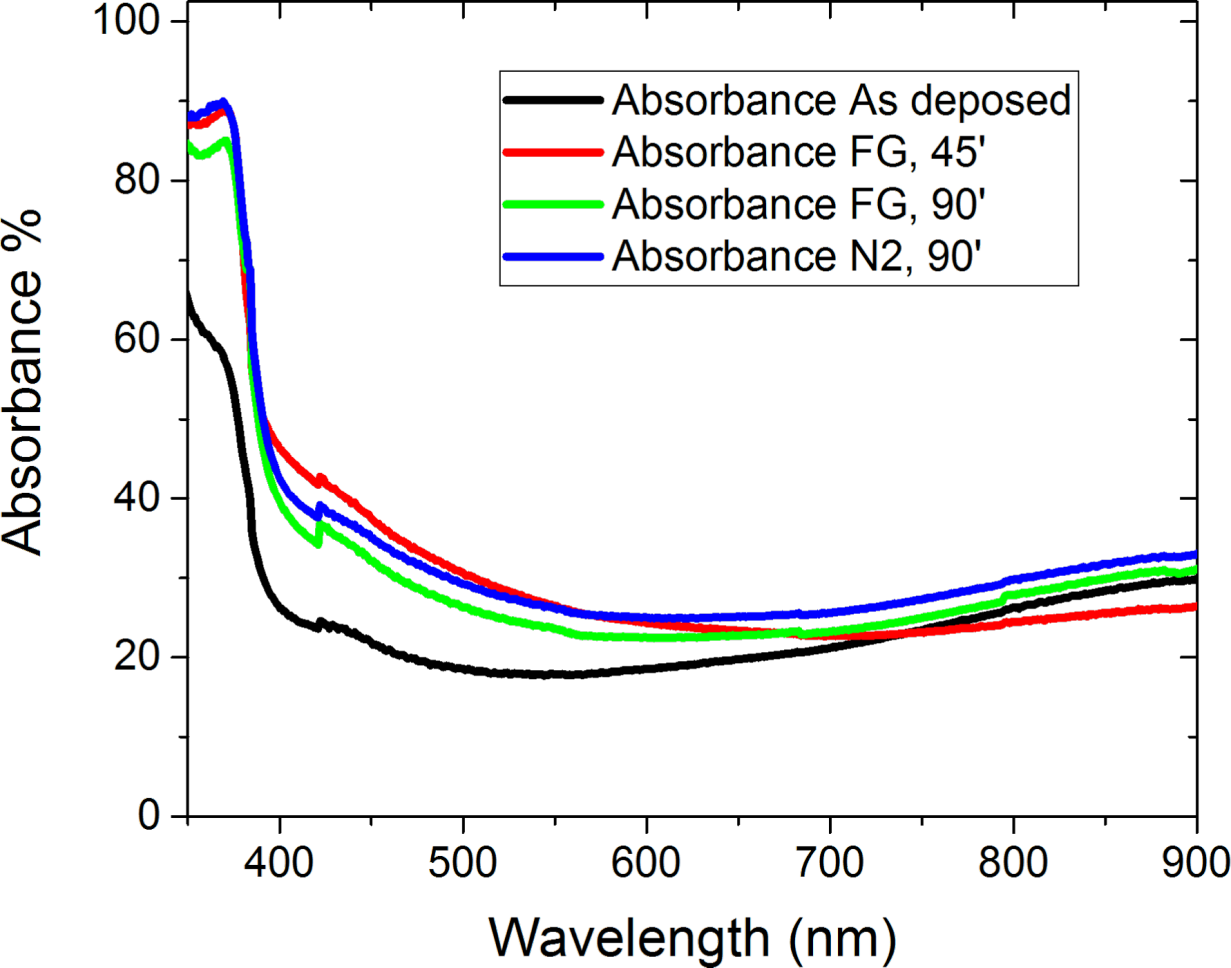
The ZnO NR absorbance measurements, as deposited in forming gas and in nitrogen for 45 minutes and 90 minutes growth time, annealed at 200 °C.

Figure 9 shows the ZnO NR layer absorbance in different conditions of the annealing gas and the growth time annealed at 200 °C. We see that the sample without annealing gas has the lowest absorbance, which implies the necessity of the annealing step. The sample, annealed in FG and grown for 45 minutes, has the highest absorbance in the region between 300 nm and 600 nm compared to those annealed in the same gas condition. However, the growth time influences the absorbance, which indicates that thin ZnO NR layers absorb more in this region.

**Figure 10:**
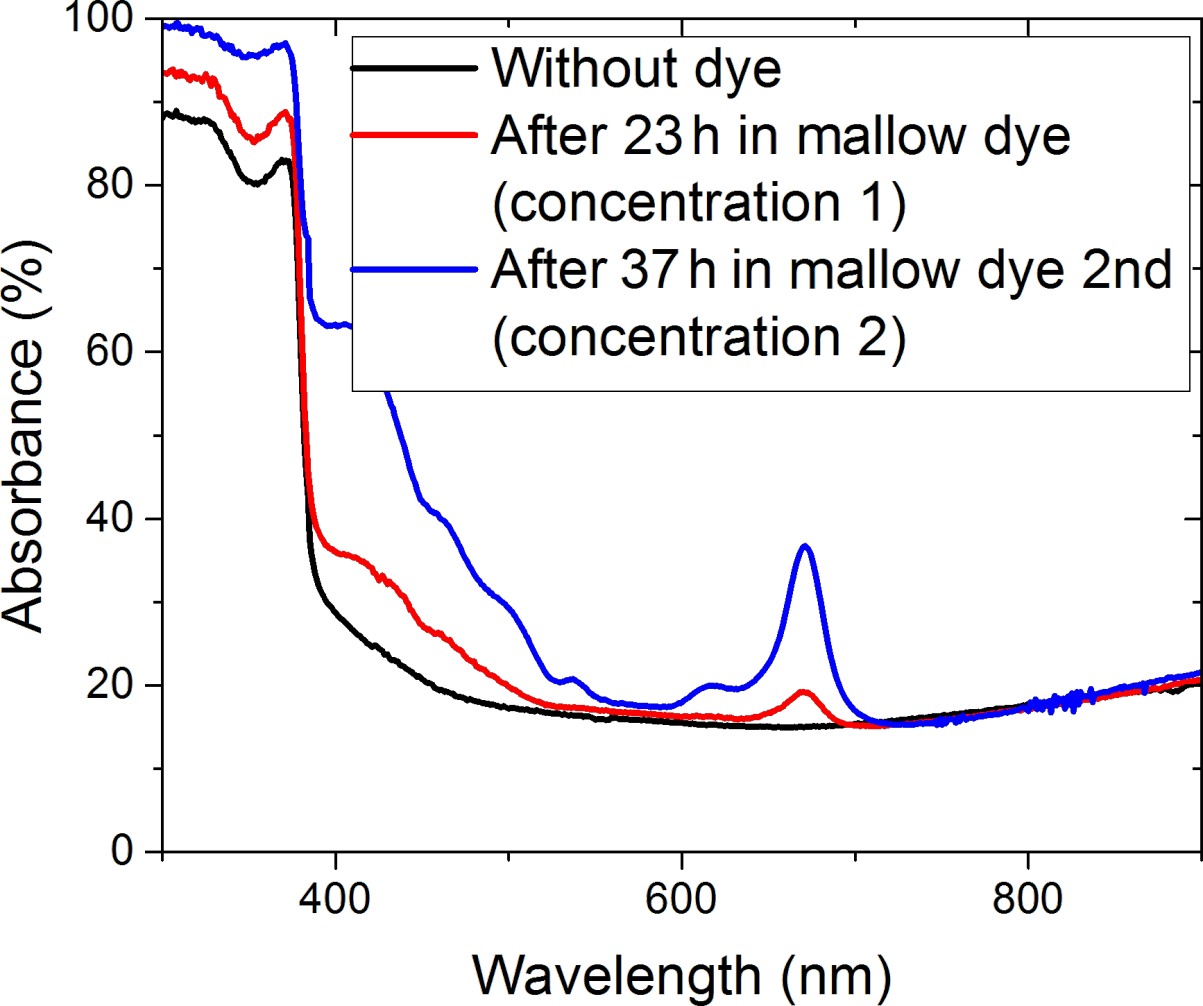
The ZnO NR absorbance measurements before and after immersion in dye for 23 h, in the first concentration, and after 37 h in the second concentration.

The second UV–vis measurements were performed after the immersion of the ZnO NR layer in dye. Figure 10 demonstrates the absorbance spectra versus the variation in time and the concentration of mallow dye. The initial concentration is 3.25 g of mallow powder stirred in 40 mL of ethanol. By multiplying the concentration three times, we increase the loaded dye in the ZnO NR layer and we clearly observed the absorbance peak related to the collected dye in the photoanode side at 665 nm.

The prepared photoanode was assembled with a platinized counter electrode. The current–voltage (*I*–*V*) measurements were carried out using a Keithley 4200 device under AM1.5 conditions.

**Figure 11:**
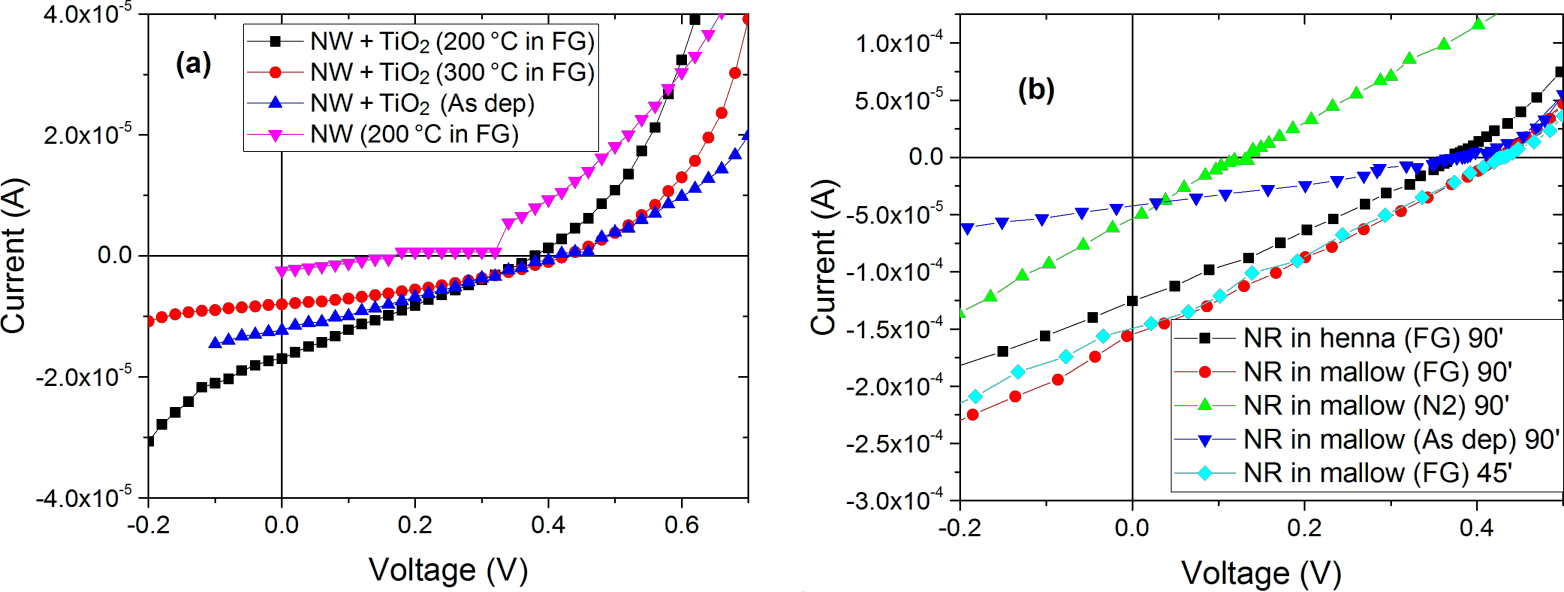
Current–voltage measurements (a) assembled cells with ZnO NWs as a layer in the photoanode side with and without a TiO_2_ blocking layer annealed at 200 °C, 300 °C, and without annealing using different ambient gas conditions. (b) Assembled cells with NR layer grown in 90 minutes and 45 minutes, annealed in FG, N_2_ and as deposited in 200 °C.

Figure 11a shows the *I*–*V* measurement response of the DSSC cells assembled with a ZnO NW layer on photoanodes annealed at different temperatures with two different structures formed by using only ZnO NWs in the photoanode and with applying a TiO_2_ blocking layer deposited by sputtering (Table 1). The mallow dye was chosen because of its optimum absorbance in the visible region. The DSSC, based on NWs with a TiO_2_ blocking layer annealed at 200 °C in FG, presents the highest *V*_OC_ compared to the NW cell without a blocking layer prepared under the same thermal conditions, where the *V*_OC_ increases from 0.169 to 0.380 V. This result verifies the main role of the blocking layer in decreasing the electron–hole recombination and the negative shift of the conduction band edge due to the increased electron density in TiO_2_, as previously reported by Yao et al. [26].

We also noticed that the increase of the temperature treatment from 200 °C to 300 °C in FG maintains the same sheet resistance of the NW layer at 5.3 Ω/sq. However, the short circuit current density of the cell decreases from 1.70 × 10^−2^ mA/cm^2^ to 8.02 × 10^−3^ mA/cm^2^. This decrease can be explained by the fact that when increasing the annealing temperature, the stability of the ZnO nanoparticles was considerably affected, which resulted in higher reaction with the dye solution.

However, the DSSC assembled without an annealed photoanode side gave a slightly higher short circuit current and smaller variation of 5% in the FF as compared to the cell annealed at 300 °C using a TiO_2_ blocking layer as shown in Figure 11. This increase can be explained by the high variation in the sheet resistance of the ZnO NWs in the photoanode side [28].

For the NR-based photoanode cells, we annealed using different gas sources. The forming gas provided the most conductive layers as compared to N_2_ and O_2_, as discussed above. Moreover, the sheet resistance increased from 5.3 × 10^3^ Ω/sq, when annealing in FG, to 90 × 10^3^ Ω/sq in N_2_. It also increased considerably in O_2_ to reach 1.4 × 10^3^ Ω/sq due to the ZnO ionization, which decreased the volume of Zn interstitials, resulting in a decrease of the carrier concentration [29].

Therefore, by comparing the two used sensitizers, under the same thermal conditions (200 °C) and at the same growth time (90 minutes), we noticed that the electrical performance was almost identical and they have the same FF value equal to 27%. By changing the growth time from 90 to 45 minutes, the FF value rises slightly from 27% to 29%. This increase implies that the reduction in the length of NRs, caused by the decrease of growth time, augments the internal shunt resistance of the cell, resulting in a small decrease of the short circuit current, *J*_sc_, from 0.155 mA/cm^2^ to 0.149 mA/cm^2^ and a small increase of *V*_OC_ from 0.425 to 0.428 V [30].

The efficiency of the ZnO NR- and NW-based DSSCs is lower than that of conventional DSSCs. The main reason for this difference in performance is related to the nature of the photosensitizer used in the present investigation.

Furthermore, the small thickness of the ZnO NRs and NWs can be a major reason for this degradation in the efficiency. However, the chemical reaction of the ZnO nanostructures when immersed in dye solution was carried out at the surface of the layer, resulting in Zn–dye complex aggregates affecting the electron injection efficiency [31].

## Conclusion

In this work, we reported the fabrication and characterization of natural DSSCs with two different ZnO layer structures. We analyzed two natural dye sensitizers extracted from henna and mallow plants in different concentrations and at various immersion times. Furthermore, we analyzed the properties of the different used dyes and we reported the most anchoring-dominant molecules. By studying different gases used in the annealing process, we noted that the FG gave the most conductive layers compared to N_2_ or O_2_, which was confirmed in the photovoltaic parameters of the final cells. We also highlight the fact that the use of a TiO_2_ blocking layer in the structure ZnO NW/TiO_2_/dye increases the photovoltaic performance of the realized DSSC by enhancing the morphological and physical properties of the photoanode.

## Experimental

Both ZnO NRs and NWs were grown on FTO substrates (1.5 × 1.5 cm^2^) by CBD [8,22]. Before the ZnO NR preparation, a seed layer of ZnO crystallites was deposited by spin coating (1000 rpm, 60 s) using a solution of 5 mM zinc acetate dihydrate in ethanol, followed by 20 min annealing in air on a hot plate (at nominal 240 °C).

However, in the case of the ZnO NWs, the seed layer was replaced by an Al film (100 nm thick). The basic Al films were sonicated in soapy water, water, ethanol and acetone before being used. After the pre-layer deposition, we prepared the CBD bath [8,22] by mixing two solutions: a 100 mL solution of variable hexamethylenetetramine (HMTA) concentration in deionized (DI) water was well stirred and preheated at 90 °C. Then, it was added to a 100 mL solution of zinc nitrate hexahydrate in DI water.

The zinc nitrate concentration in the CBD bath was fixed at 25 mM both for ZnO NR and NW growth. The HMTA final concentration was set at 25 mM and 50 mM for three different growth times (45 min, 60 min and 90 min) in case of ZnO NRs. In the synthesis of NWs, the growth time was set to 5 min. The HMTA concentration was 25 mM. Moreover, 16 mM of ammonium hydroxide (NH_4_F) was added to the synthetic solution since its presence enhanced the quality of the NWs [22].

The DSSC preparation started by covering an active area of 0.5 × 0.5 cm^2^ TCO glass with a thin film ZnO layer (NRs, NWs, or TiO_2_ on NWs). After the annealing step, the prepared film was submerged in a solution containing the natural dye. The used natural dyes were prepared using ethanol as the solvent. The electrode and the counter electrode were sealed with thermoplastic sealing film (Meltonix 1170-60PF from SOLARONIX), followed by injection of (Iodolyte AN-50) the electrolyte (SOLARONIX).

The used henna and mallow powders were prepared in-house by drying henna and mallow plants. Afterwards, the dried plants were milled and sieved to obtain the final powder.

Current–voltage characteristics and layer conductivity were measured using a computer-controlled Keithley 4200 instrument under standard conditions using a solar simulator based on a 150 W xenon lamp equipped with an ASTM-certified filter with a calibrated Si photodiode to produce the AM1.5 solar spectrum.

For the FTIR measurements, we used a Nicolet 560 FTIR spectrometer with a measurement region varying between 400 and 4000 cm^−1^ with suitable scan resolution of 2 cm^−1^. The UV–vis measurements were studied by means of a UV–vis Lambda 40 spectrophotometer with an integrating sphere. The study of the structural properties of ZnO NWs and NRs was performed using scanning electron microscopy (Gemini field emission SEM, Carl Zeiss SUPRATM 25).
